# A conscious choice: Is it ethical to aim for unconsciousness at the end of life?

**DOI:** 10.1111/bioe.12838

**Published:** 2020-12-17

**Authors:** Antony Takla, Julian Savulescu, Dominic J. C. Wilkinson

**Affiliations:** ^1^ Faculty of Medicine, Nursing and Health Sciences, Monash University Clayton Victoria Australia; ^2^ Oxford Uehiro Centre for Practical Ethics Faculty of Philosophy University of Oxford Oxford United Kingdom of Great Britain and Northern Ireland; ^3^ Murdoch Children’s Research Institute Melbourne Victoria Australia; ^4^ John Radcliffe Hospital Oxford United Kingdom of Great Britain and Northern Ireland

**Keywords:** doctrine of double effect, end of life care, sedation, terminal anaesthesia, terminal sedation, unconsciousness

## Abstract

One of the most commonly referenced ethical principles when it comes to the management of dying patients is the doctrine of double effect (DDE). The DDE affirms that it is acceptable to cause side effects (e.g. respiratory depression) as a consequence of symptom‐focused treatment. Much discussion of the ethics of end of life care focuses on the question of whether actions (or omissions) would hasten (or cause) death, and whether that is permissible. However, there is a separate question about the permissibility of hastening or causing unconsciousness in dying patients. Some authors have argued that the DDE would not permit end of life care that directly aims to render the patient unconscious. The claim is that consciousness is an objective human good and therefore doctors should not intentionally (and permanently) suppress it. Three types of end of life care (EOLC) practices will be explored in this article. The first is symptom‐based management (e.g. analgesia); the second is proportional terminal sedation as a means of relieving suffering (also referred to as palliative sedation or continuous deep sedation); and finally, deliberate and rapid sedation to unconsciousness until death (a practice we call terminal anaesthesia in this paper). After examining the common arguments for the various types of symptom‐based management and sedation, we apply the DDE to the latter two types of EOLC practices. We argue that aiming at unconsciousness, contrary to some claims, can be morally good or at least morally neutral in some dying patients.

## 1 SETTING THE SCENE

*Mr Holmes is a 75‐year‐old man who suffers from colorectal cancer that, despite treatment with surgery and chemotherapy, has metastasized to his lungs and liver. He is admitted to hospital for increasing shortness of breath and abdominal pain from his liver lesions. Physician A sees Mr Holmes and thinks that he probably has a week or two of life left. Mr Holmes expresses a wish to have palliative care. He is afraid of being in pain and asks that he be made as comfortable as possible over that period, even if it comes at the cost of his consciousness. The physician starts him on a morphine infusion*.

## 2 THE DDE

The doctrine of double effect (DDE), originally formulated by Thomas Aquinas in the *Summa Theologica*, aims to distinguish between outcomes that are intended by an agent, and outcomes that are merely side‐effects—perhaps foreseen, but not intended.^1^ It also places an emphasis on the moral nature of the action itself, in that one cannot achieve desired ends, even if they are good, using immoral means.^2^ According to a standard formulation of the DDE, an action is permissible if and only if the following criteria are met:

1. The action itself is not bad (it must be good or morally neutral).
2. Only the good effect is intended, and the bad effect is not.
3. The bad effect is not part of the means needed to achieve the good effect (the bad effect is merely an outcome of the means used).
4. The good effect outweighs the bad effect (they are proportionate).

According to this doctrine, a physician may administer high doses of pain‐relieving medications, such as morphine, at the potential cost of hastening death, as long as the physician’s intention is the relief of suffering, not the accelerating of death.^3^ In contrast to this, the DDE suggests that doctors must not carry out an act like assisting suicide or performing euthanasia, because even if the main intention is relief from suffering, physicians must not use a ‘bad’ means, (such as death), to attain the desired outcome.^4^ (Whether it is necessarily bad to aim at death is debatable, but beyond the scope of this paper. Here, we merely highlight how the doctrine has been used.)

The DDE does not only feature in ethical debates but has also made its way into the legal realm: physicians in the UK and the US have been acquitted from charges of causing death because their primary intentions were to alleviate the suffering of their dying patients.^5^ The main rulings from these various court cases were that if the physician acted with good intentions to provide relief from suffering for their patient, but incidentally hastened death, then they were not acting out of line with their duties as physicians.^6^

However, although the DDE is often invoked to justify or reject certain medical treatments (such as terminal sedation), it is not without its shortcomings, and not all ethicists agree on its usefulness and applicability.^7^ For example, some have argued that it is not always feasible (for others or even oneself) to fully know one’s intentions and that in many cases, physicians may have multiple intentions at once^8^: they are not so easy to categorize as the doctrine requires. Nonetheless, the DDE is still widely used in both the ethical and legal realms and so we will review its application to various EOLC practices, as well as the relationship between the depth of sedation and the potential hastening of death.

Ethicist Daniel Sulmasy has recently argued that although consciousness is not a ‘transcendental value’, it is still an objective human good and should always be viewed as a ‘true cost’ when physicians diminish it.^9^ Based on this view, he contends that whilst gradual, or ‘parsimonious’ sedation can sometimes be acceptable, directly aiming at unconsciousness in dying patients is never permissible.^10^ Sulmasy’s paper provided a rich and detailed analysis, including his own account of the philosophy of medical therapeutics.^11^ We will focus on some of the key arguments relating to consciousness and the care of the dying and will also present our own perspectives where our views diverge from his.

## 3 THREE DIFFERENT WAYS THAT SEDATION COULD RESULT FROM OR BE CAUSED BY TREATMENT

### 3.1 Sedation as a side‐effect of analgesia

One of the most common medications used in dying patients, especially for cancer‐related pain, are opioids.^12^ Drugs like morphine, fentanyl or oxycodone are potent analgesics and frequently cause reduced consciousness as a side‐effect. A Cochrane review looking at the prevalence of altered consciousness because of opioid use found that 23% of end‐of‐life‐care patients on opioids experience somnolence or drowsiness.^13^

In the case of opioids, administering the drug is not a morally bad action in and of itself—it is widely accepted that opioids may be used for patients in pain. The good effect that is intended is pain relief, and the bad effect is potentially altered or reduced consciousness. (Whether reduced consciousness is a bad effect or not will be explored in greater detail soon.) Given that reduced consciousness is not part of the means used (that is, morphine doesn’t have its analgesic function through reducing consciousness) then criterion three of the DDE is adequately met. Finally, the benefit of a dying patient being pain‐free generally outweighs the reduced or altered consciousness—this is precisely the evaluation made by Mr Holmes in the example.

It is therefore straightforward and uncontroversial that reduced consciousness caused as a side‐effect of analgesia could be justified by the DDE in dying patients.

### 3.2 Proportional terminal sedation

*Mr Holmes has been on the morphine infusion for the past three days but has inadequate symptom control despite increasing doses. His physician decides to add midazolam to the morphine infusion. Midazolam is a sedating benzodiazepine. Physician A plans to increase the midazolam gradually until Mr Holmes appears comfortable, even if that means sedating him to unconsciousness*.

The use of titrated sedation in patients with treatment‐refractory symptoms at the end of life is often referred to as terminal sedation. The goal of terminal sedation is to alleviate suffering by reducing consciousness. If necessary, patients are deeply sedated, even to the point of unconsciousness, and remain so until death.

Would this be ethical? Some have maintained that the DDE can justify the use of a sedative in this case.^14^ On that view, administering sedatives is not, in itself, a bad action. Titration to symptoms is essential so as to not breach criterion four of the DDE and ensure that the good effect of being sedated (and therefore not aware of one’s suffering) is adequately weighed up against the ‘bad’ effect of hastening death, which can occur from using sedatives unsparingly. If the physician’s intention is the relief of suffering rather than hastening death, then they are not acting out of line with the DDE. This line of argument, however, presupposes that diminished consciousness can be classified as a good outcome (at least in this circumstance) and that sedation in these circumstances is a permissible action.

Contrary to this view, Sulmasy has argued that proportional terminal sedation (he calls it parsimonious direct sedation) is not consistent with the DDE. One of Sulmasy’s key objections is his argument that ‘sedation is not a human good’ (p. 250).^15^ Sulmasy claims that it is never justifiable to relieve suffering by rendering a patient unconscious until death (p. 238). Even if the physician’s intentions were the relief of suffering, the means by which this is carried out in terminal sedation (via reduced consciousness) is bad, and so one would fail to meet the first condition of the DDE.^16^ If Sulmasy’s argument is accepted, terminal sedation would not be justifiable, even if it had no impact on the timing on death. If terminal sedation *were* to hasten death, however, the implication of Sulmasy’s argument is that physicians could not draw on the DDE as an ethical or legal defence.^17^ (Sulmasy accepts that in ‘extremely rare circumstances’, sedation at the end of life might be justified using a different ethical rationale—the canon of parsimony, which he defines as using only ‘as much therapeutic force as necessary’ to achieve a desired goal).^18^

Nonetheless, one initial reply to Sulmasy’s argument might distinguish between sedation and unconsciousness. After all, sedation is widely accepted in medicine where patients are anxious or experiencing something very unpleasant but unavoidable. For example, patients receive pre‐medication to reduce anxiety prior to surgical or dental procedures, or conscious sedation for procedures like the reduction of a fracture or a colonoscopy. Unconsciousness, in the case above, is an unfortunate side effect of Mr Holmes’ sedation, arising only if his symptoms cannot be controlled any other way. However, this sort of argument would not apply in cases of end of life care where unconsciousness is clearly the primary aim of treatment. Consider the following alternate scenario.

### 3.3 Terminal anaesthesia

*Upon receiving the news that he has a week to two of life left, Mr Holmes expresses anxiety about suffering symptoms of pain, discomfort and delirium over the coming days. He has a long‐standing history of insomnia. Mr Holmes has no close family or friends. He requests to receive sufficient sedation that he is deeply asleep and does not wish to wake again. Mr Holmes is given a combination of morphine and low dose Propofol. He falls rapidly unconscious and remains so for the ensuing 5 days, before quietly passing away*.

The above practice is sometimes referred to as ‘rapid sedation to unconsciousness’. Sulmasy refers to it as ‘sedation to unconsciousness and death’. 19 We will call it ‘terminal anaesthesia’ (TA) to better capture the concept that its main goal is to rapidly achieve deep unconsciousness for a dying patient. TA, therefore, like surgical general anaesthesia, does not titrate to the smallest possible dose, but directly uses a dose that is known to, or expected to, bring total unconsciousness for the patient (this stands in contrast to proportional terminal sedation whereby the main goal is alleviating suffering by carefully titrating sedatives). TA would not involve using enormous doses that are intended to hasten death; rather, enough to cause unconsciousness without, where possible, causing undue or avoidable suppression of other vital functions like cardiorespiratory function.

In terminal anaesthesia, it is clear that unconsciousness is not a side effect of treatment. It is the aim of treatment. It is important, then, to determine whether this can be a permissible goal for a physician.

## 4 THE VALUE OF CONSCIOUSNESS

One of the concerns about applying the DDE to sedation at the end of life is based on the claim that consciousness is an objective human good that ought to not be actively diminished or removed.^20^ Of course, consciousness is *usually* objectively good, but there may be circumstances where consciousness is no longer good for a patient. If this can be the case, then reducing or removing consciousness, in certain circumstances, can be a good outcome to aim at.

Consciousness, *tout court* (without further qualification), enables humans to flourish.^21^ It is through consciousness that people are able to experience themselves, others and the world around them. Indeed, without consciousness, we would not be able to experience ‘freedom, love, aesthetic experiences, spirituality, reason, morality, humour’ etc.^22^ In general, loss of consciousness ought to be viewed as a misfortune that medicine should aim to correct or reverse. However, *pace* Sulmasy, that does not always apply. The value of consciousness for patients at the end of their life can be reduced.

One way of defending terminal anaesthesia would be to argue that *mere* consciousness is not of intrinsic value. It is the contents of consciousness that make it either good or bad. Experiences of love, pleasure, beauty, etc. are good, while experiences of pain, suffering, isolation, loneliness, etc. are bad. Imagine that a creature were conscious but their experiences were completely bland and neutral—they had neither positive nor negative experiences. If consciousness is only instrumentally valuable, there would be no positive or negative value for this individual. On that view, the value of consciousness cannot be evaluated separately from its contents. Unconsciousness would be, by definition, value‐neutral.

However, we do not need to establish whether mere consciousness has intrinsic value or is instrumentally valuable. It is enough to grant that consciousness can be diminished by disease and its contents can be bad. Furthermore, bad consciousness can outweigh good consciousness at the end of life.

Consider again Mr Holmes. Either because of the opioids he would be receiving, or from liver failure, renal failure and subsequent encephalopathy, he may be delirious and/or agitated, as often can be the case in the final weeks or days of life. A systematic review of eight studies found that 58.8 to 88% of inpatients receiving palliative care experience delirium in their final hours to weeks before death.^23^ That state of delirium is a serious compromise in one’s consciousness. Patients are no longer able to relate to themselves, others, or the world around them in a meaningful way. In fact, delirium is often a distressing experience. The patient is confused as to whom they are, who the people around them are, and where they themselves are in time and space. In those instances, patients may not be able to experience reason, love, spirituality or any of the aforementioned benefits of consciousness as they normally would. Being conscious, for that person, is not necessarily objectively, or subjectively, good. This may be even more likely in patients with neurodegenerative illnesses whereby physical brain changes progressively attenuate consciousness, especially towards the end of their life.

This is not to say that all patients are precluded from having any meaningful experiences whilst receiving end of life care. Delirium, for example, may be better on some days or at certain times of the day, allowing for some meaningful experiences. However, those periods of attenuated symptoms are likely, in general, to get shorter and less frequent as a patient nears their death.

**FIGURE 1:**
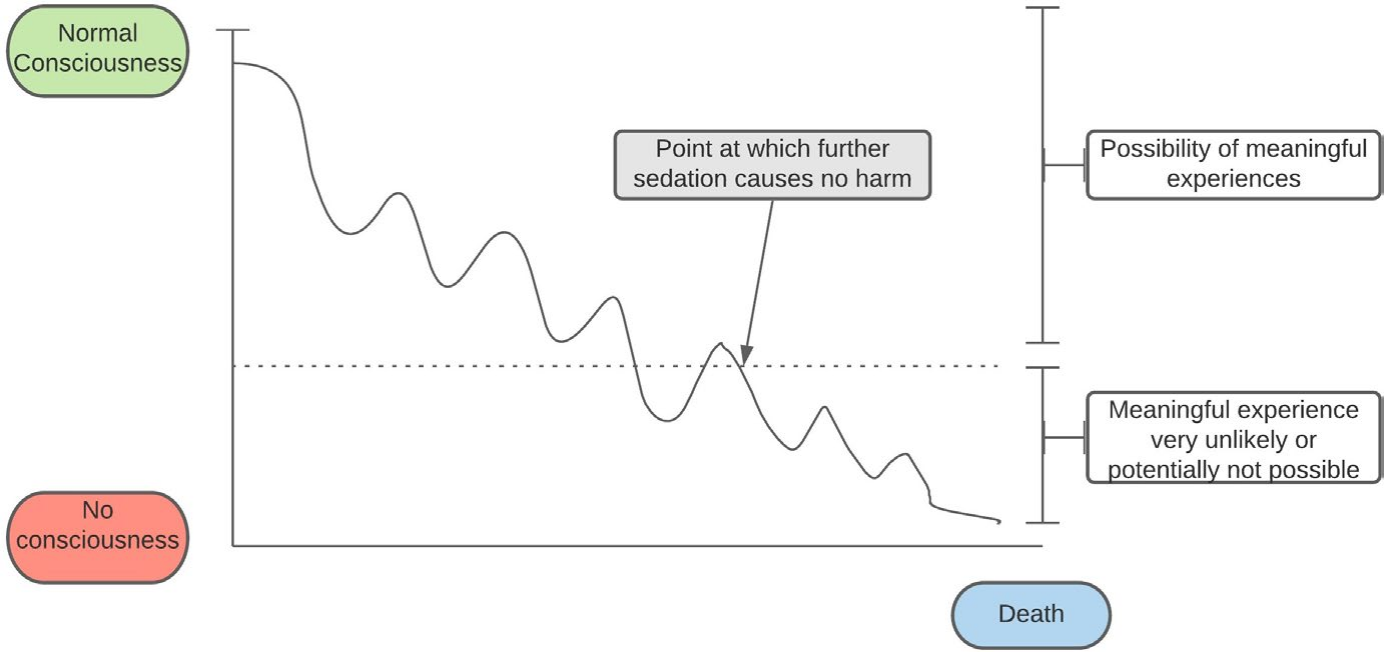
Harms caused by sedation [Colour figure can be viewed at wileyonlinelibrary.com]

Consciousness lies on a spectrum. For dying patients for whom consciousness is already compromised in ways that prevent them from engaging in meaningful experiences (either from the disease itself, or its management through opioids or other medications), further sedation is, at worst, neutral. Although deeper sedation potentially attenuates valuable experiences more than lighter sedation, there is a point of impaired consciousness beyond which a patient can no longer meaningfully engage in any activity. Beyond that point, further sedation is not any more harmful or bad from the patient’s perspective. The diagram in Figure 1 seeks to capture the point we are trying to make.

Of course, the point in time after which consciousness cannot (or is very unlikely to) entail meaningful experience is not easy to determine. This should be discussed with the patient (or the patient’s family if discussion with the patient is not possible) and a value judgement is made, based on the patient’s values, on whether a physician may commence deeper sedation at the cost of waiving potential valuable/meaningful moments in the future.

To reiterate, this is not to say that consciousness at the end of life always lacks value. There can be meaningful, reconciliatory, spiritual or personal experiences that make consciousness desirable. But where consciousness is compromised by disease, its symptoms or its management, this no longer always holds. Therefore, aiming to render a patient less conscious, is ethically neutral where:

1. the patient is at the end of their life, and is either already on, or expected to need, large doses of medications that are likely to tamper with their consciousness (opioids and benzodiazepines) in ways that rob it of its goodness; or
2. the patient’s consciousness is already compromised because of their disease process and further reductions in consciousness are not likely to cause further harm at this point.

One important question is at what point consciousness is of no further net value. Sulmasy accepts that in circumstances where a patient’s consciousness has been completely overwhelmed by their symptoms, that there may be no barrier to sedation since there is ‘effectively, no consciousness to be lost’. (p. 259)^24^ This seems to us too extreme. (After all, if there is no consciousness to be lost, there would be no point to administering sedation.) A more charitable interpretation of that argument is that consciousness (at least in a literal sense) still remains but is so wholly consumed by suffering that no good can come from sustaining it. Ultimately, there is a judgement that will need to be made about the value of further conscious experience. The important ethical question is who should decide.

What if Mr Holmes were currently fully aware? On Sulmasy’s view, his consciousness would be intrinsically valuable. However, we would argue that the value of consciousness is not something that someone else (a physician, say) can or should solely dictate. In a dying patient, the degree of suffering, combined with their personal dispositions and intentions, will determine whether to them, consciousness is a good or a bad thing. Some patients may desire to remain conscious and aware of the presence of their loved ones for as long as possible and wish to cherish any final experiences. For them, consciousness is a good that needs to be preserved if possible. Sigmoid Freud had this view. He famously chose to be able to think clearly, though in pain with terminal throat cancer, rather than experience diminished consciousness.^25^

Others, however, can reach a point in a terminal illness where, faced with imminent and inevitable demise, they have no desire for further conscious experiences. This would clearly be a rational view if consciousness has instrumental value and is a means by which individuals realize their desires and intentions, be those relational, spiritual or existential. However, a desire to no longer be conscious can also be rationalized on the view that consciousness has intrinsic value in a restricted sense. This is to say, it can have intrinsic positive and negative value. It is good overall when the positive aspects of consciousness outweigh the bad, rather than good in and of itself irrespective of what kind of experiences one is having. For those who have no further desires because of suffering towards the end of life, all things considered, consciousness can become deeply undesirable and not an objective human good.

Some may reject this restricted view of the value of consciousness. That may mean that, for themselves, they would choose to forswear terminal sedation at the end of life. However, the view that consciousness has restricted value is a plausible and reasonable alternative perspective. It is difficult to see why the view that consciousness is always valuable should be imposed on people who do not share it.

One setting where it is widely accepted that consciousness can lack value (and unconsciousness be ethically desirable) is during a surgical procedure. We normally think that it is ethically acceptable for patients to choose to have an operation under general anaesthesia, even if the procedure could be performed safely and comfortably under local anaesthetic (e.g. wisdom teeth extractions). Patients should be free to make these decisions, based on their weighing up of potential pain and anxiety related to the procedure against the risks of general anaesthesia. Being unconscious for a procedure is obviously different in one way: there is a restorative end in sight (that is, anaesthesia is a means to an end and is not permanent).^26^ Nonetheless, there is a valuable comparison to be made. Patient values should be elicited and respected for terminal anaesthesia just as they are for anaesthesia during surgery. While for the dying patient, death is imminent and inevitable, the end does not have to dictate the process. Just because there is no restorative end to the dying process, it does not mean that the dying patient must be obliged to remain conscious and aware. They should not be forced to embrace the experience of dying any more than a dental patient has to embrace and accept the experience of a dental extraction or a mother has to embrace the pain of childbirth. If the dying process is deemed insufferable and undesirable by the patient, they should have the same access as non‐dying patients to medicines and medical expertise that could blunt their negative experiences.

A clear and unambiguous request by a patient to be made unconscious may not always be possible, especially if the patient’s capacity to make those decisions is compromised by their disease, its management or the distress they might be in. Although not the topic of this paper, these decisions are best discussed and noted down formally in advance, before the patient is in their dying moments. A proxy can also be used to ascertain what the patient would have wanted (weighing up potential suffering with potential meaningful experiences on the patient’s behalf). Ultimately, this should be a dynamic process between the physician, the patient and their family. If the patient or their family repeatedly express a desire for unconsciousness (and the patient is indeed at the end of their life), then the physician should grant them that request as long as the patient is not known to be at a particularly high risk of reacting adversely to anaesthesia.

If our view of consciousness is accepted, then the DDE can be applied and serve as an ethical justification for both terminal sedation and terminal anaesthesia. There are circumstances when using sedation is indeed not intrinsically bad, thus satisfying the first criterion of the DDE. The physician’s intention is to reduce (or remove) consciousness, which is a desired ‘good’ outcome (where consciousness is judged by the patient to have little positive value after weighing up potential meaningful experiences with current or potential suffering), and the bad side effect is hastening of death. The bad effect (death) is not part of the means used to achieve the good effect. And finally, the proportionality criterion can be fulfilled: there are at least some situations where the wish to be ‘asleep’ or unconscious (and thus guaranteed to be free from suffering) outweighs the risk of hastening death.

## 5 OBJECTIONS

We will consider three key objections to the view that we have outlined above.

### 5.1 Equivalence of terminal anaesthesia and euthanasia

Arguments around terminal sedation overlap with those relating to euthanasia at the end of life. Some may feel that the practices are so similar that terminal sedation or anaesthesia represent a form of euthanasia. Sulmasy claimed that the arguments that justify sedation to unconsciousness and death (what we have called terminal anaesthesia) would also justify euthanasia (p. 260).^27^ He implied (by a form of modus tollens) that if euthanasia is rejected then anaesthesia at the end of life must also be rejected.

It is beyond the scope of this paper to address the permissibility of euthanasia. However, we will highlight some conceptual reasons why we believe that it would be coherent for individuals or jurisdictions to distinguish between the two.

1. We have tried to show that there are different reasonable views about the value of consciousness. Some people may view consciousness as instrumentally valuable, and others may view it as intrinsically valuable but in a restricted, non‐absolutist sense, in which case it may be ok to aim at unconsciousness, but not at death. Sulmasy himself agrees that consciousness is different from life in that it is not a ‘transcendental value’.^28^ We think this is a distinction that may be shared by many.
2. Although there may be some cases where differentiating terminal anaesthesia and euthanasia is difficult, the two practices are conceptually distinct. Anaesthesia is reversible, while euthanasia (by definition) is not. Let’s say that the treating physician makes a mistake about the prognosis of a dying patient and anaesthetises them because they believe they will die imminently. The doctors could stop the infusion of anaesthetic used and this patient would regain consciousness again if needed.
3. Anaesthesia or sedation in dying patients would not (necessarily) cause death. It is conceivable that death would be hastened in some patients who receive terminal anaesthesia. But that will depend on both the cases selected and the agents and doses used. It is not necessarily the case that this would occur. There are studies reporting the use of propofol in end of life care whereby unconsciousness was rapidly achieved (at an anaesthetic depth) and patients remained on the infusion for up to 14 days.^29^ Other studies observe that deep sedation commenced in patients with a short life expectancy has minimal influence on the timing of death.^30^ Guidelines often restrict deep sedation to patients who are expected to die imminently (at most, within two weeks) so that the provision or withholding of artificial nutrition and hydration (ANH) will not contribute to the patient’s death.^31^
4. Permanent unconsciousness is not the same as death. While there are ongoing philosophical debates about the criteria for determination of death, there are no jurisdictions that equate permanent loss of consciousness alone with death.^32^ Patients who are in a permanent vegetative state are treated completely different from patients who have died. One key distinction, however, is that patients in a vegetative state have lost the anatomical or functional capacity to ever regain consciousness again, whereas that is not the case for a patient who is receiving sedation/anaesthesia.^33^
5. Finally, there are the legal and social aspects of the two practices. Euthanasia is illegal in most countries and remains controversial and debated even where it is lawful in limited circumstances. Some patients (for example because of their religious beliefs) may decline euthanasia at the end of life. However, they may accept analgesia and sedation. General anaesthesia is widely legally and socially accepted to treat pain for medical procedures. It is permitted by all major religions. In this paper we have likened the dying process to a ‘procedure’; one that can involve considerable pain and anxiety. It is normal medical practice to use medicines to blunt some of those negative experiences. One need not challenge the legal system or society’s perspectives on the role of the physician in order to permit sedation or anaesthesia at the end of life.

We agree with Sulmasy that the request for euthanasia and anaesthesia at the end of life may sound very similar: ‘Doctor just put me to sleep’. Both actions may indeed be motivated by dissociating a person from their suffering, given that at the point when this request is made, the physician can no longer remove the suffering itself. However, that does not mean, as we have highlighted, that the actions themselves are morally or conceptually the same.

### 5.2 ‘Terminal sedation’ for those who are not dying

Some may suggest that if the judgement about the benefits and burdens of consciousness are deferred to the patient, that this could mean that patients who could survive for a long time (perhaps even without a physical illness) might choose to be sedated or anaesthetized to unconsciousness. This might be offered as a slippery slope objection to terminal sedation/anaesthesia (or a reductio ad absurdum).

It is beyond the scope of this paper to set out what the pre‐conditions should be for terminal sedation. (In a separate paper we propose that terminal anaesthesia should only be an option for patients predicted to die within 2 weeks.)^34^ However, paying attention to a patient’s wishes and values does not mean that any patient request will be followed.

The reason is essentially the same reason that doctors do not give opioids to every patient with pain: it is a benefit‐risk assessment. For a dying patient, death is imminent. The main consideration for them is how much they value their consciousness and whatever experiences it still allows them to have. For a non‐dying patient, there is an objective risk of causing death when it would not have otherwise occurred at that time. Doctors are justified in not providing morphine to patients who have a stubbed toe—even if the patient judges the risk/benefit acceptable.

Secondly, we do not claim that suffering is of no value at all. Indeed, suffering can build resilience, perseverance, and many other positive character traits that can serve the individual in other aspects of life. It is not the role of medicine to relieve or obliterate any and all suffering whenever the patient so desires. However, for a dying patient, the potential positives from their suffering will reasonably depend on their worldview. If the patient wishes to remain conscious for existential, spiritual or reconciliatory benefits, that ought to be granted and respected. On the other hand, if the patient’s worldview does not include such benefits, then it is hard to see how they benefit from being conscious as they die.

### 5.3 Proportionate sedation preferable

We have argued that it would be ethical to aim directly at unconsciousness in a dying patient (where this is consistent with their wishes). However, some may claim that to be ethically proportionate, the lowest possible doses must be used. (One proposed additional condition for the DDE is that agents should strive to minimize the foreseen harm.)^35^ This may mean that terminal sedation but not anaesthesia is permissible.

However, there are some reasons to reject this argument. In brief, empirical studies raise doubt about how pain‐free a sedated patient truly is when they appear so to the physician.^36^ Studies in surgical anaesthesia show that even where the patient is thought to be completely unconscious, a small percentage of patients still experience intra‐operative awareness that they cannot communicate.^37^ If this were the case with anaesthesia, then it is much more likely that some palliative care patients who receive minimal and parsimonious sedation could still be experiencing suffering (that they are not able to communicate). In addition, for proportional terminal sedation to work, a physician has to wait for a patient to evince distress or suffering before the dose can be increased. This may mean periods of time when the patient is suffering until the ‘optimum’ dose is achieved—although the previous argument raises doubt about how to assess when this is ever truly achieved.

Some patients may prefer proportionate sedation at the end of life—to maximize their awareness and minimize the risk of either complete unconsciousness or hastening death. However, other patients may see no value in remaining conscious, and experience considerable anxiety at the thought of experiencing pain in their dying minutes or hours (perhaps especially if they are unable to communicate this). The question of ethical proportionality in end of life care must ultimately be based on the values of the patient, not those of the attending physician.

## 6 CONCLUSION

In this paper, we have focused on the ethics of causing unconsciousness at the end of life. Human consciousness is good but not always or universally so. For most people, and for most of our life, consciousness is good because it brings with it fulfilling experiences and human flourishing. At the end of life, consciousness is often fragile and compromised. For some, there may come a time when its goodness or usefulness subsides.

We have argued that intentionally causing unconsciousness can be both ethical and compatible with the doctrine of double effect. We rejected the view that consciousness is intrinsically valuable in an unrestricted sense and unconsciousness always bad. Consciousness in dying patients can already be compromised to an extent where it no longer provides the patient with valuable experiences, rendering further sedation morally neutral. Consciousness may also be unwanted and even feared in a dying patient, since the patient has a desire not to suffer, and has no remaining desires to remain awake and aware. In these circumstances, removing consciousness is not inherently bad (it is at worst morally neutral), and the DDE can therefore be used to justify its use. These arguments provide a defence for sedation at the end of life even where this comes at some risk of hastening death. However, they also support the view that in select cases there may be an ethical role for general anaesthesia in dying patients.

## CONFLICT OF INTEREST

The authors declare no conflict of interest.

## FUNDING INFORMATION

The authors were supported by grants from the Wellcome Trust WT203132 (JS, DW) WT104848 (JS). JS, through his affiliation with the Murdoch Children’s Research Institute was supported by the Victorian Government’s Operational Infrastructure Support Program. The funders had no role in the preparation of this manuscript or the decision to submit for publication.

## Footnotes

^1^
McIntyre, A. (Spring 2019 Edition). "Doctrine of Double Effect". The Stanford Encyclopedia of Philosophy. E. N. Zalta (Ed.). Retrieved from: https://plato.stanford.edu/entries/double‐effect/

^2^
Ibid.

^3^
Whether morphine does hasten death or not, is beyond the scope of this paper but we note in passing that some studies suggest that morphine does not necessarily hasten death and therefore invoking the DDE may be unnecessary. Sykes, N., & Thorns, A. (2003). The use of opioids and sedatives at the end of life. *Lancet Oncology, 4*(5), 312–318; Sterckx, S., & Raus, K. (2014). Continuous sedation at the end of life. *The Oxford handbook of ethics at the end of life* (pp. 1–19). Younger S., & Arnold, R (eds.) Oxford Handbooks Online. 10.1093/oxfordhb/9780199974412.013.7. Retreived from: https://www.oxfordhandbooks.com/view/10.1093/oxfordhb/9780199974412.001.0001/oxfordhb-9780199974412-e-7

^4^
Sterckx & Raus, op. cit. note 3. p.1‐19.

^5^
Ibid.

^6^
Davies, M. (1998). *Textbook on medical law* (2nd ed.). London, UK: Blackstone; Vacco v. Quill. 1997. 521 U.S. 793 (U.S. Supreme Court); Washington v. Glucksberg. 1997. 521 U.S. 702 (US Supreme Court).

^7^
Riisfeldt, T. (2018). Weakening the ethical distinction between euthanasia, palliative opioid use and palliative sedation. *Journal of Medical Ethics, 45*(2), 125–130.10.1136/medethics-2018-10507430352790

^8^
Ibid; Janssens, R., van Delden, J. J. M., & Widdershoven, G. A. M. (2012). Palliative sedation: Not just normal medical practice. Ethical reflections on the Royal Dutch Medical Association’s guideline on palliative sedation. *Journal of Medical Ethics, 38*(11), 664‐668.10.1136/medethics-2011-10035322811556

^9^
Sulmasy, D. P. (2018). The last low whispers of our dead: When is it ethically justifiable to render a patient unconscious until death? *Theoretical Medicine and Bioethics, 39*(3), 233–263.10.1007/s11017-018-9459-730132300

^10^
Ibid.

^11^
Ibid.

^12^
Wiffen, P. J., Derry, S., & Moore, R. A. (2014). Impact of morphine, fentanyl, oxycodone or codeine on patient consciousness, appetite and thirst when used to treat cancer pain. *Cochrane Database of Systematic Reviews. 5*, CD011056.10.1002/14651858.CD011056.pub2PMC648354024874470

^13^
Ibid.

^14^
Quill, T. E., Lo, B., Brock, D. W., & Meisel, A. (2009). Last‐resort options for palliative sedation*. Annals of Internal Medicine, 151*, 421–424; Rousseau, P. C. (2002). Palliative sedation. *American Journal of Hospice & Palliative Care, 19*(5), 295–297.10.7326/0003-4819-151-6-200909150-0000719755367

^15^
Sulmasy, op. cit. note 9. p.233‐263.

^16^
Ibid.

^17^
Ibid; Sterckx & Raus op. cit. note 3. p.1‐19.

^18^
Sulmasy, op. cit. note 9. p.233‐263. The question of whether sedation hastens death is outside the scope of this paper. Nevertheless, several studies have shown that there is no significant difference in survival time between sedated and non‐sedated patients receiving end of life care, and that sedation is unlikely to hasten death if used on patients who are already in their active dying phase. Maltoni, M., Pittureri, C., Scarpi, E., Piccinini, L., Martini, F., Turci, P., …. Amadori, D. (2009). Palliative sedation therapy does not hasten death: Results from a prospective multi‐center study. *Annals of Oncology, 20*(7), 1163–1169; Claessens, P., Menten, J., Schotsmans, P., & Broeckaert, B. (2011). Palliative sedation, not slow euthanasia: A prospective, longitudinal study of sedation in Flemish palliative care units. *Journal of Pain and Symptom Management, 41*(1), 14–24. One important factor regarding the hastening of death is the administration or withholding of artificial nutrition and hydration (ANH). Most guidelines recommend that sedation should only be commenced in patients whose life expectancy is no more than two weeks, others more strictly limit it to days or hours so that even if ANH were withheld or ceased, it would have little to no effect on the timing of death. Sterckx & Raus, op. cit. note 3. p1‐19.

^19^
Sulmasy, op. cit. note 9. p.233‐263.

^20^
Ibid.

^21^
Ibid.

^22^
Ibid.

^23^
Hosie, A., Davidson, P. M., Agar, M., Sanderson, C. R., & Phillips, J. (2013). Delirium prevalence, incidence, and implications for screening in specialist palliative care inpatient settings: A systematic review. *Palliative Medicine, 27*(6), 486–498.10.1177/026921631245721422988044

^24^ Sulmasy, op. cit. note 9. p.233‐263

^25^
Griffin, J. (1986). *Well‐being: Its meaning, measurement and moral importance*. UK: Oxford University Press.

^26^
Sulmasy, op. cit. note 9. p263‐263.

^27^ ibid.

^28^
Ibid.

^29^
Lundström, S., Zachrisson, U., & Fürst, C. (2005). When nothing helps: Propofol as sedative and antiemetic in palliative cancer care. *Journal of Pain and Symptom Management, 30*(6), 570–577.10.1016/j.jpainsymman.2005.05.01716376744

^30^
Rietjens, J., van Delden, J., Onwuteaka‐Philipsen, B., Buiting, H., Maas, P., & Heide, A. (2008). Continuous deep sedation for patients nearing death in the Netherlands: Descriptive study. *BMJ, 336*(7648), 810–813.10.1136/bmj.39504.531505.25PMC229233218344245

^31^
Sterckx & Raus, op. cit. note 3. p1‐19.; Janssens et al., op. cit. note 8. p664‐668.

^32^
Smith M. (2012). Brain death: Time for an international consensus. *BJA: British Journal of Anaesthesia, 108*(Suppl_1), i6–i9.10.1093/bja/aer35522194433

^33^
Raho, J. A., & Miccinesi, G. (2015). Contesting the equivalency of continuous sedation until death and physician‐assisted suicide/euthanasia: A commentary on LiPuma. *The Journal of Medicine and Philosophy: A Forum for Bioethics and Philosophy of Medicine, 40*(5), 529–553. 10.1093/jmp/jhv018 26242447

^34^
Takla, A., Savulescu, J., Wilkinson, D., & Pandit, J. General Anaesthesia in End‐of‐Life‐Care. In peer‐review.10.1111/anae.15459PMC858198333878803

^35^
McIntyre, A, op. cit. note 1.

^36^
Alkire, M., Hudetz, A., & Tononi, G. (2008). Consciousness and anesthesia. *Science, 322*(5903), 876–880; Noreika, V., Jylhänkangas, L., Móró, L., Valli, K., Kaskinoro, K., Aantaa, R., … Revonsuo, A. (2011). Consciousness lost and found: Subjective experiences in an unresponsive state. *Brain and Cognition, 77*, 327–334; Sinmyee, S., Pandit, V., Pascual, J., Dahan, A., Heidegger, T., Kreienbühl, G, … Pandit, J. (2019). Legal and ethical implications of defining an optimum means of achieving unconsciousness in assisted dying. *Anaesthesia, 74*(5), 630–637.10.1111/anae.1453230786320

^37^
Blokland, Y., Farquhar, J., Mourisse, J., Scheffer, G., Lerou, J., & Bruhn, J. (2012). Towards a novel monitor of intraoperative awareness: Selecting paradigm settings for a movement‐based brain‐computer interface. *PLoS ONE, 7*(9), e44336.10.1371/journal.pone.0044336PMC343541822970202

